# Cerebellar cryptococcomas

**DOI:** 10.1007/s10072-023-06635-w

**Published:** 2023-01-26

**Authors:** Carlo Manco, Nicola De Stefano, Roberto Marconi

**Affiliations:** 1 Centre of Precision and Translational Medicine; Medicine, Surgery and Neuroscience Department, University of Siena, Siena, Italy; 2 Unit of Neurology, Cardio-Thoracic-Neuro-Vascular Department, Misericordia Hospital, Grosseto, Italy

**Keywords:** Cerebellar cryptococcomas, MRI, CSF analysis

**Fig. 1:**
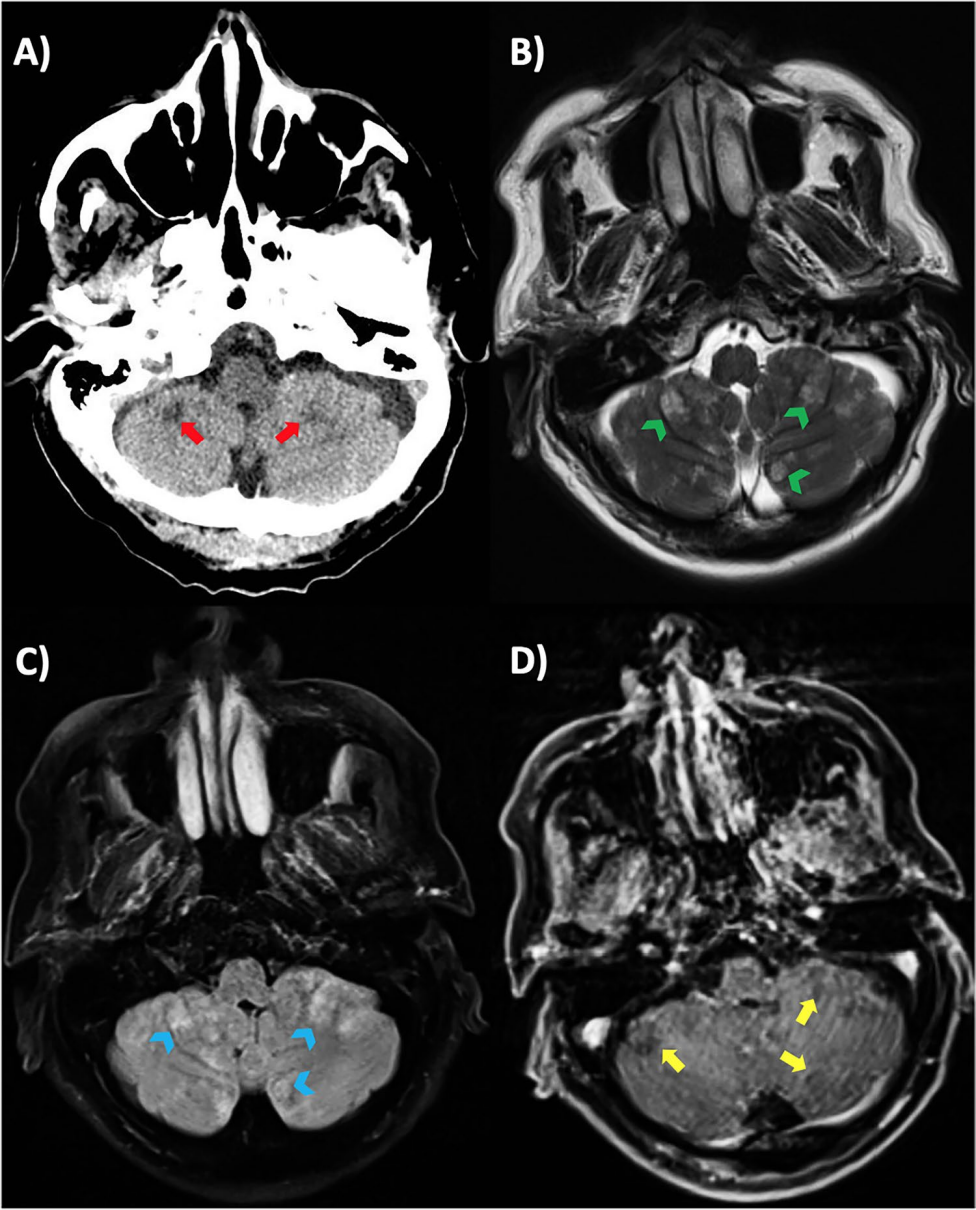
**A** CT brain showing cerebellar hypodense lesions (arrow); **B** and **C** axial T2-weighted MRI sequences showing hyperintense focal parenchymal masses (arrowheads); **D** post-gadolinium MRI sequence showing no contrast enhancement (arrows)

**Fig. 2:**
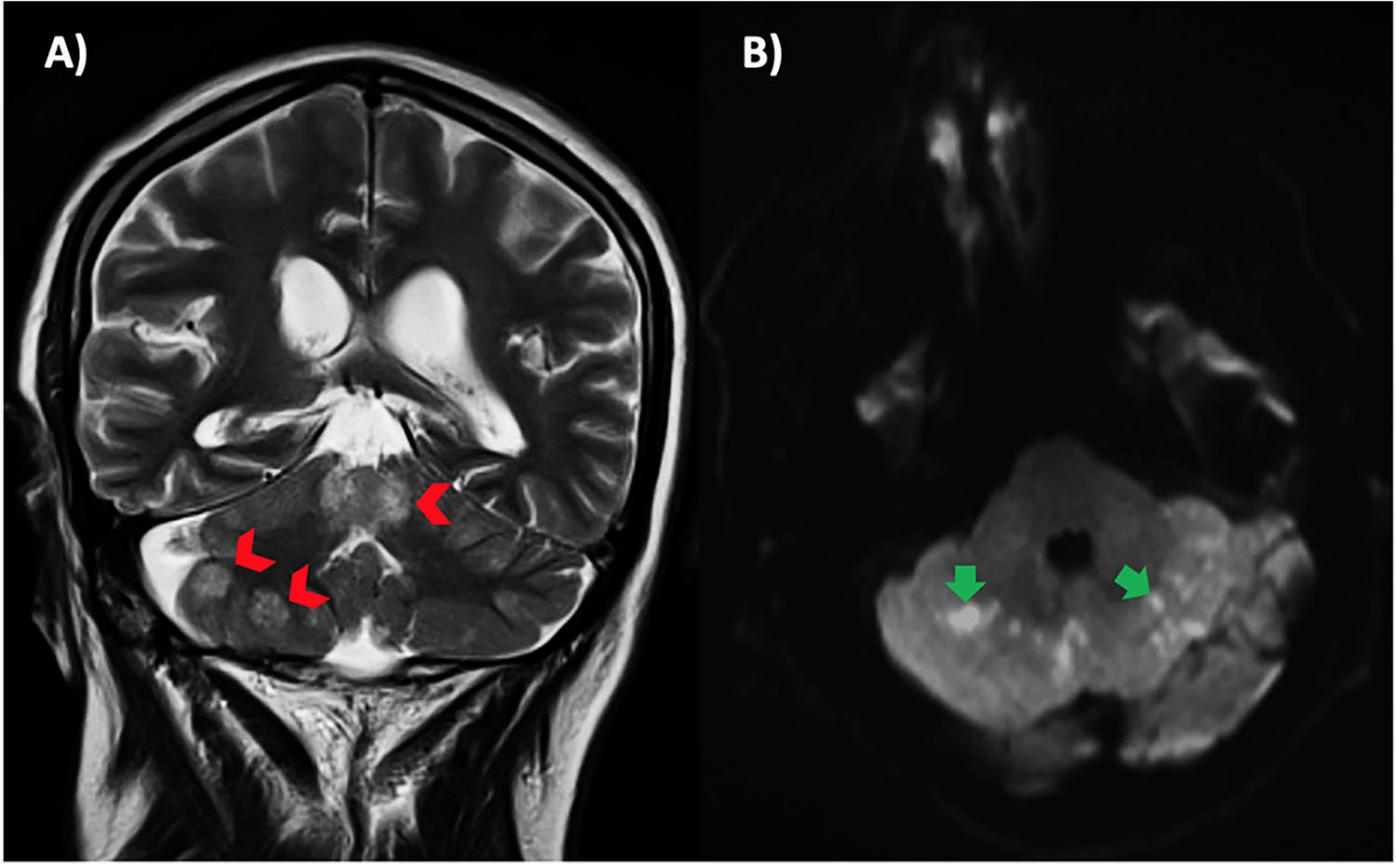
**A** Coronal slice showing exclusively cerebellar involvement (arrowheads). **B** Cerebellar diffusion restriction due to parenchymal lesions (arrows)

A 72-year-old man, trader, from Central America was admitted to the neurologic unit in Grosseto after 2 weeks onset of balance disturbances and headache. Two days before, a low-grade fever developed, and his wife brought him to the hospital. Neurological examination showed dysarthria, bilateral nystagmus, and ataxia. Brain CT showed multiple low-density non-enhancing cerebellar lesions and diffuse cerebellar edema (Fig. 1). Brain MRI revealed multiple hyperintense areas in T2-weighted sequences in the whole cerebellum, with diffusion restriction in DWI, no enhancement post-gadolinium infusion (Figs. 1 and 2).

Virological tests and HIV screening were negative. Chest-RX was unremarkable. CSF analysis documented hypoglycorrachia, hyperproteinorrachia, pleomorphic pleocytosis, and yeast cells. Film-array detected *Cryptococcus neoformans/gattii*. CSF culture and serum antigen (titer 1: 1024) were positive for *Cryptococcus neoformans*. Hematological investigations revealed idiopathic CD4 lymphocytopenia. The patient received therapy with high doses of amphotericin B and fluconazole for 2 weeks, followed by fluconazole for 6 months.

Cryptococcosis is an important infection recognized for its ability to cause meningoencephalitis, especially in immunocompromised hosts, although it can occur in immunocompetent hosts [1].

Risk factors for cryptococcosis are HIV infection, diabetes, and idiopathic CD4 lymphocytopenia; in many cases, infection occurs through inhalation of the microorganism [1, 2]. A focal parenchymal mass known as a cerebral cryptococcoma may follow disseminated infection with Cryptococcus spp; in 73% of cases, it is associated with edema and is most frequently localized in the basal ganglia, thalamus, and cerebellum [2]. Symptoms such as headache, fever, or mental status changes may appear initially and be sneaky but should warrant diagnostic testing [1]. Brain CT can help detect cryptococcomas although its sensitivity is lower than that of brain MRI [1]. Cerebral cryptococcomas can be a diagnostic challenge and are often confused at neuroimaging with dilated perivascular spaces, embolic stroke, and inflammatory or malignant processes [2]. DWI abnormalities likely reflect cellular infiltration and the presence of high protein fluid and have been described in fungal cerebritis [3]. The lesions’ little or no contrast enhancement in MRI can be a valid aid in the differential diagnosis of tumors or inflammatory processes, which are usually associated with uptake contrast lesions.

The right neuroradiological framework can point to appropriate diagnostic tests, favoring a timely antifungal therapy.
